# Association between LMP2/LMP7 genetic variability and cancer susceptibility, especially among Asians: evidence from a meta-analysis

**DOI:** 10.18632/oncotarget.18752

**Published:** 2017-06-28

**Authors:** Yang Wu, Dong-Fang Liu, Jing-Jing Zhang, Xiao Li, Zi-Peng Lu, Guo-Dong Shi, Hao Yuan, Yu-Gang Ge, Peng-Fei Wu, Yan Wang, Kui-Rong Jiang, Yi Miao

**Affiliations:** ^1^ Pancreas Center, The First Affiliated Hospital of Nanjing Medical University, Nanjing, 210000, China; ^2^ Pancreas Institute, Nanjing Medical University, Nanjing, 210000, China; ^3^ Department of General Surgery, The First Affiliated Hospital of Nanjing Medical University, Nanjing, 210009, China; ^4^ Department of Urology, The Affiliated Cancer Hospital of Jiangsu Province of Nanjing Medical University, Nanjing, 210009, China; ^5^ The Endoscopy Center, The First Affiliated Hospital of Nanjing Medical University, Nanjing, 210009, China

**Keywords:** LMP2, LMP7, polymorphism, meta-analysis, cancer

## Abstract

Low molecular mass protein (LMP) gene performs a critical role in the foreign antigen processing machine via the major histocompatibility complex-I (MHC-I) complex CD8+ cytotoxic T lymphocytes (CTL) pathway. Recent studies have reported the association of LMP2-60 G>A (rs17587) and LMP7-145 C>A (rs2071543) polymorphisms with various types of cancers, but the outcomes remained inconsistent. To obtain a reliable conclusion, we summarized available data and conducted a meta-analysis involving a total of 19 published studies. Evidences were obtained from the PubMed, Google Scholar, Web of Science and Chinese National Knowledge Infrastructure (CNKI) databases. The results demonstrated that the rs17587 and rs2071543 polymorphisms were associated with an increased cancer risk in the recessive and homozygote models. Stratified analyses by ethnicity indicated a significant association only in Asian population. Furthermore, rs17587 showed a greater susceptibility to gynecological cancers, while rs2071543 increased the risk of gastrointestinal and gynecological cancers. Our results indicate that the LMP2 rs17587 and LMP7 rs2071543 polymorphisms may act as risk factors for cancer, especially for Asian populations. Additional larger-scale multicenter studies should be performed to validate our results.

## INTRODUCTION

Currently, cancer is a major cause of human death and a public health problem that seriously threatens human health worldwide [1]. Large epidemiological and biological investigations have indicated that genetic and environmental factors contribute to tumourigenesis. However, the exact mechanisms of carcinogenesis have not been fully illuminated.

In recent years, many researches have confirmed that the elimination of cancer cell is promoted by the classical MHC-I-restricted T lymphocytes pathway [2, 3]. In this pathway, the CD8+ CTL could recognize the peptide antigen of cancer cell which is processed by LMP2/LMP7 molecules (GenBank Accession: X66401.1 GI: 34634) and presented by TAP1/TAP2 molecules (TAP, transporter associated with antigen presentation) [4]. The LMP/TAP system could recognize cancer antigen and act a pivotal role in immune surveillance via MHC-I molecule and CTL in the human host protective immunity [5, 6]. Therefore, the integrality of LMP2/LMP7 function played a restrictive effect on the processing of cancer cell antigen.

Previous researches indicated that the polymorphisms of LMP2- 60(G>A) and LMP7-145(C>A) would give rise to the functional alteration, and then impaired the capacity of antigen processing machine. As a consequence, LMP2/LMP7 gene variants were associated with development, occurrence and prognosis of many types of cancers, such as colorectal cancer, cervical cancer, gastric carcinoma and so on [7-16]. However, no consensus has yet been achieved, which was partially due to heterogeneity between cancer types, different ethnicities of patient cohorts, diverse genotyping methods and relatively small sample sizes. Accordingly, we conducted this meta-analysis to derive a more precise and up-to-date estimation of associations of LMP gene polymorphisms with cancer risk.

## RESULTS

### Literature search and study characteristics

**Figure 1:**
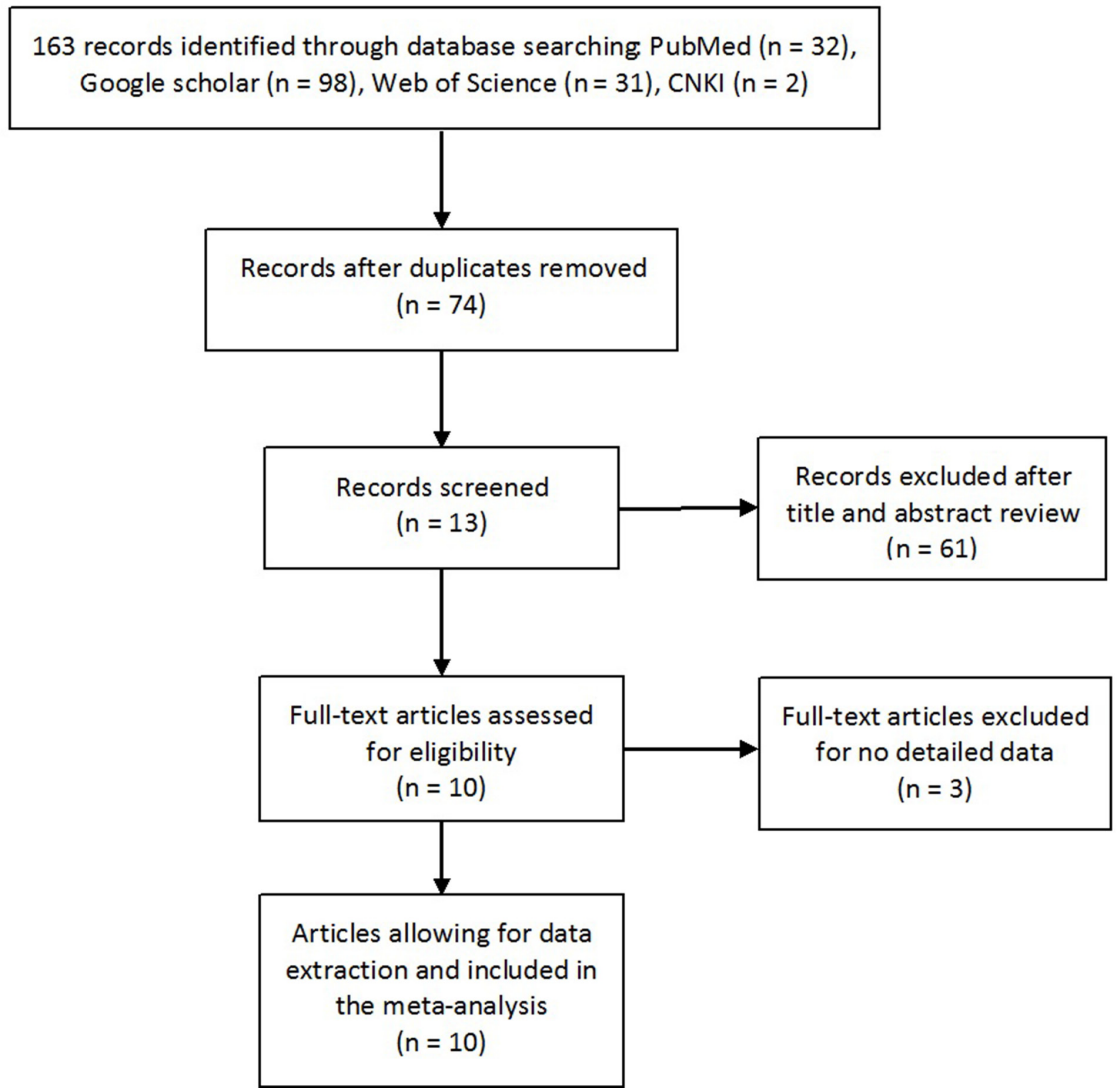
The flow diagram of retrieval for this study CNKI: China National Knowledge Infrastructure.

The flow chart of study selection process is shown in Figure 1. We identified 163 records, among which 10 publications appeared to be eligible and were retrieved in full texts [7-16]. Eventually, a total of 19 case-control studies from 10 publications (4360 cases and 4987 controls) were included in this study, and details of each study were recorded in Table 1. The eligible studies presented data for several different cancer types, including gastric cancer, cervical cancer, ovarian cancer, hematological malignancy, colorectal cancer and esophageal squamous cell carcinoma. Among these 19 studies, 11 were based on Asian populations [7-9, 14-16], 8 on Caucasian populations [10-13]. Furthermore, the genotyping methods utilized in the studies included PCR-RFLP (RFLP, restriction fragment length polymorphism), Taqman, Sequencing and ARMS-PCR (ARMS, amplification refractory mutation system). Quality assessment with Newcastle-Ottawa Scale (NOS) showed that 17 studies had a quality score higher than 7 points, and 2 studies had a quality score equal to 7 points (Table 1).

**Table 1:** Characteristics of studies included in the meta-analysis

SNP	Study	Ethnicity	Genotyping method	Source of control	Cancer type	Case			Control			P value of HWE	NOS points
						AA	AB	BB	AA	AB	BB		
LMP2 rs17587	Ma(2015)	Asian	PCR-RFLP	HB	GC	338	151	13	335	155	12	0.228	8
	Mehta(2015)	Asian	Taqman	PB	CC	82	90	21	79	77	10	0.117	9
	Song(2014)	Asian	PCR-RFLP	HB	OC	129	72	34	208	86	44	0.000	8
	Gerceker(2013)	Caucasian	PCR-RFLP	PB	HM	46	52	34	49	46	35	0.001	9
	Fellerhoff(2011)	Caucasian	ARMS-PCR	HB	CRC	87	71	16	86	68	11	0.617	8
	Deshpande(2008)	Caucasian	Sequencing	PB	CC	206	90	21	246	155	16	0.162	9
	Mehta(2007)	Caucasian	Taqman	HB	CC	66	52	9	65	54	5	0.126	8
	Cao(2006)	Asian	PCR-RFLP	PB	GC	93	47	5	95	55	2	0.053	7
	Cao(2005)	Asian	PCR-RFLP	PB	ESCC	167	83	15	239	102	16	0.234	8
LMP7 rs2071543	Ma(2015)	Asian	PCR-RFLP	HB	GC	310	169	23	349	141	12	0.612	8
	Mehta(2015)	Asian	Taqman	PB	CC	173	18	1	141	22	1	0.888	9
	Song(2014)	Asian	PCR-RFLP	HB	OC	120	86	29	249	76	13	0.243	8
	Gerceker(2013)	Caucasian	PCR-RFLP	PB	HM	111	18	3	112	17	1	0.692	9
	Fellerhoff(2011)	Caucasian	ARMS-PCR	HB	CRC	97	70	7	145	20	0	0.407	8
	Deshpande(2008)	Caucasian	Sequencing	PB	CC	268	60	2	351	69	1	0.208	9
	Mehta(2007)	Caucasian	Taqman	HB	CC	96	31	0	78	43	3	0.297	8
	Yang(2007)	Asian	PCR-RFLP	PB	ESCC	137	25	6	194	84	5	0.228	8
	Cao(2006)	Asian	PCR-RFLP	PB	GC	63	69	13	59	85	8	0.001	7
	Cao(2005)	Asian	Sequencing	PB	ESCC	130	114	21	210	130	17	0.583	8

PCR: polymerase chain reaction; RFLP: restriction fragment length polymorphism; ARMS: amplification refractory mutation system; HB: hospital-based studies; PB: population-based studies. GC: gastric cancer; CC: cervical cancer; OC: ovarian cancer; HM: hematological malignancy; CRC: colorectal cancer; ESCC: esophageal squamous cell carcinoma; HWE: Hardy–Weinberg equilibrium, P > 0.05 indicates that the participants in the control group met the HWE. A: the major allele; B: the minor allele; NOS: Newcastle-Ottawa Scale.

### Meta-analysis of the LMP polymorphisms and cancer risk

**Figure 2:**
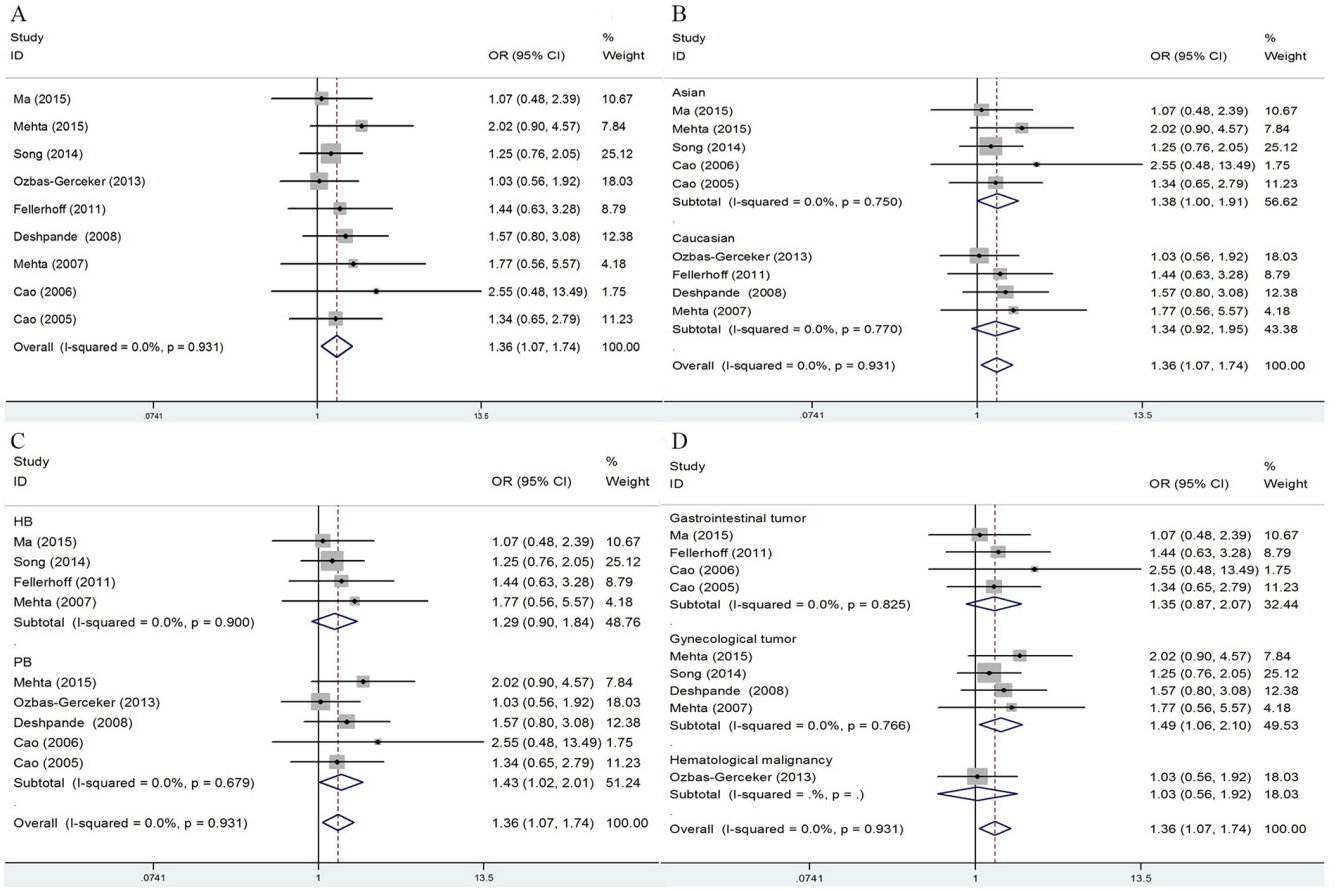
Forest plots of cancer risk associated with LMP2-60 G>A (rs17587) polymorphism under homozygote model **(A)** Overall result; **(B)** subgroup analysis by ethnicity; **(C)** stratified analysis by design of study; **(D)** subgroup analysis by cancer types.

The main results of this meta-analysis are listed in Table 2. Nine studies involving 2,090 cases and 2,351 controls were included for rs17587. As indicated in Figure 2 and Table 2, we observed an increased cancer susceptibility associated with the rs17587 polymorphism under homozygote and recessive models [AA vs. GG: OR = 1.36, (95% CI: 1.07-1.74), *P* = 0.931, *I*^*2*^ = 0.0%; AA vs. GA/GG: OR = 1.31; (95% CI: 1.03-1.65), *P* = 0.787, *I*^*2*^ = 0.0%]. Subgroup analysis by ethnicity showed a significant association only in the Asian populations [AA vs. GG: OR = 1.38, (95% CI: 1.00-1.91), *P* = 0.750, *I*^*2*^ = 0.0%] (Figure 2B, Table 2). In addition, stratified analysis by design of study showed a significant relationship in population-based studies [AA vs. GG: OR = 1.43, (95% CI: 1.02-2.01), *P* = 0.679, *I*^*2*^ = 0.0%; AA vs. GA/GG: OR = 1.38; (95% CI: 1.00-1.90), *P* = 0.446, *I*^*2*^ = 0.0%] (Figure 2C, Table 2). In the cancer-specific analysis, the results showed significant correlations between rs17587 and risk of gynecological cancers in different comparison models. [AA vs. GG: OR = 1.49, (95% CI: 1.06-2.10), *P* = 0.766, *I*^*2*^ = 0.0%; AA vs. GA/GG: OR = 1.46; (95% CI: 1.05-2.03), *P* = 0.572, *I*^*2*^ = 0.0%] (Figure 2D, Table 2).

**Table 2:** Meta-analysis results of association between LMP2/LMP7 polymorphisms and cancer risk

		LMP2-60 G>A (rs17587)					LMP7-145 C>A (rs2071543)			
		AA vs GG		AA vs GG/GA			AA vs CC		AA vs CC/CA	
Variables	N	OR(95%CI)	*P*/*I*^*2*^(%)	OR(95%CI)	*P*/*I*^*2*^(%)	N	OR(95%CI)	*P*/*I*^*2*^(%)	OR(95%CI)	*P*/*I*^*2*^(%)
Total	9	**1.36(1.07-1.74)**	0.931/0.0	**1.31(1.03-1.65)**	0.787/0.00	10	**2.38(1.72-3.29)**	0.215/24.8	**2.17(1.57-2.99)**	0.506/0.00
**Ethnicity**										
Asian	5	**1.38(1.00-1.91)**	0.750/0.00	1.31(0.95-1.79)	0.705/0.00	6	**2.37(1.68-3.36)**	0.344/11.2	**2.16(1.54-3.04)**	0.679/0.00
Caucasian	4	1.34(0.92-1.95)	0.770/0.00	1.31(0.92-1.86)	0.466/0.00	4	2.39(0.93-6.13)	0.096/52.6	2.20(0.83-5.83)	0.161/41.7
**Source of control**										
PB	5	**1.43(1.02-2.01)**	0.679/0.00	**1.38(1.00-1.90)**	0.446/0.00	6	**1.83(1.14-2.93)**	0.976/0.00	**1.82(1.15-2.88)**	0.987/0.00
HB	4	1.29(0.90,1.84)	0.900/0.00	1.23(0.87-1.74)	0.850/0.00	4	**3.00(1.90-4.73)**	0.032/65.9	**2.54(1.62-4.00)**	0.087/54.4
**Cancer type**										
Gastrointestinal	4	1.35(0.87-2.07)	0.825/0.00	1.33(0.87-2.03)	0.808/0.00	5	**2.15(1.44-3.20)**	0.517/0.00	**2.02(1.36-2.99)**	0.706/0.00
Gynecological	4	**1.49(1.06-2.10)**	0.766/0.00	**1.46(1.05-2.03)**	0.572/0.00	4	**2.91(1.62-5.21)**	0.070/57.4	**2.46(1.38-4.40)**	0.153/43.0
Hematological	1	-	-	-	-	1	-	-	-	-

N: number of studies; P: P value of Q test for heterogeneity; PB: population-based; HB: hospital-based.

**Figure 3:**
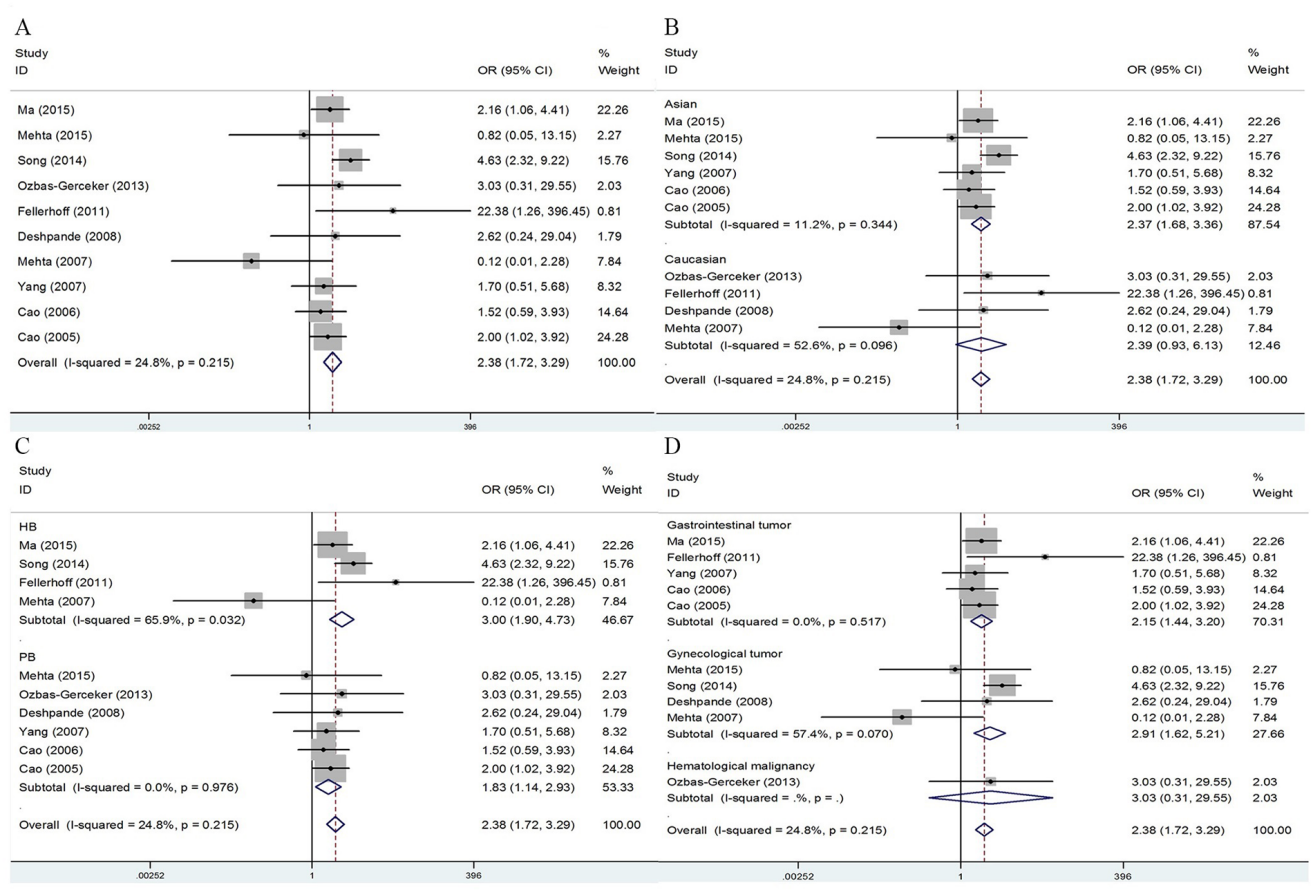
Forest plots of cancer risk associated with LMP7-145 C>A (rs2071543) polymorphism under homozygote model **(A)** Overall result; **(B)** subgroup analysis by ethnicity; **(C)** stratified analysis by design of study; **(D)** subgroup analysis by cancer types.

The association of LMP7 rs2071543 polymorphism with cancer risk was investigated in 10 studies involving 2,270 cases and 2,636 controls. This polymorphism was associated with an increased cancer susceptibility in the overall population under the two models [AA vs. CC: OR = 2.38, (95% CI: 1.72-3.29), *P* = 0.215, *I*^*2*^ = 24.8%; AA vs. CC/CA: OR = 2.17, (95% CI: 1.57-2.99), *P* = 0.506, *I*^*2*^ = 0.0%] (Figure 3A, Table 2). In the ethnicity-specific analysis, a significant association was observed in the Asian populations [AA vs. CC: OR = 2.37, (95% CI: 1.68-3.36), *P* = 0.344, *I*^*2*^ = 11.2%; AA vs. CC/CA: OR = 2.16, (95% CI: 1.54-3.04), *P* = 0.679, *I*^*2*^ = 0.0%], but not in the Caucasian populations (Figure 3B, Table 2). Additionally, subgroup analysis by the source of control showed a significant correlation in population-based and hospital-based studies (Figure 3C, Table 2). Furthermore, in analysis stratified by cancer types, the results showed significant correlations between rs2071543 and the risk of gastrointestinal cancers [AA vs. CC: OR = 2.15, (95% CI: 1.44-3.20), *P* = 0.517, *I*^*2*^ = 0.0%; AA vs. CC/CA: OR = 2.02, (95% CI: 1.36-3.04), *P* = 0.679, *I*^*2*^ = 0.0%] and gynecological cancers [AA vs. CC: OR = 2.37, (95% CI: 1.68-3.36), *P* = 0.344, *I*^*2*^ = 11.2%; AA vs. CC/CA: OR = 2.16, (95% CI: 1.54-3.04), *P* = 0.679, *I*^*2*^ = 0.0%] (Figure 3D, Table 2).

### Test of heterogeneity

In this study, Chi-squared-based Q-statistic test was used to evaluate between-study heterogeneity that resulted from methodological or clinical dissimilarity across studies. As a result, heterogeneity between studies was not identified for rs17587 and rs2071543 under the homozygote and recessive models (*P* > 0.1) (Table 2).

### Sensitivity and publication bias analysis

**Figure 4:**
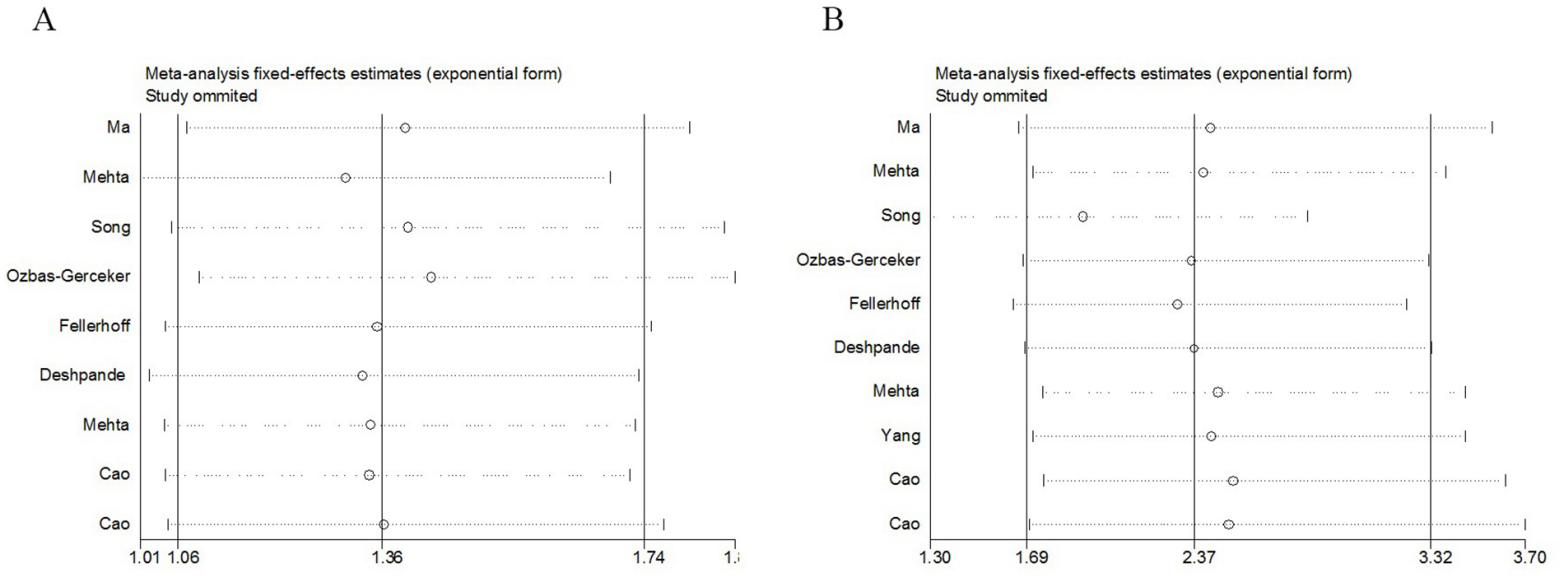
Sensitivity analysis of cancer risk associated with rs17587 **(A)** and rs2071543 **(B)** polymorphisms under homozygous model.

To confirm the reliability of results, we further conducted sensitivity analyses by repeating the meta-analysis while sequentially omitting the studies included (one deleted at a time). The analysis outcomes demonstrated that no single study greatly influenced the overall cancer risk estimations with respect to the LMP polymorphisms. (Figure 4A–4B), suggesting that our results were robust.

**Figure 5:**
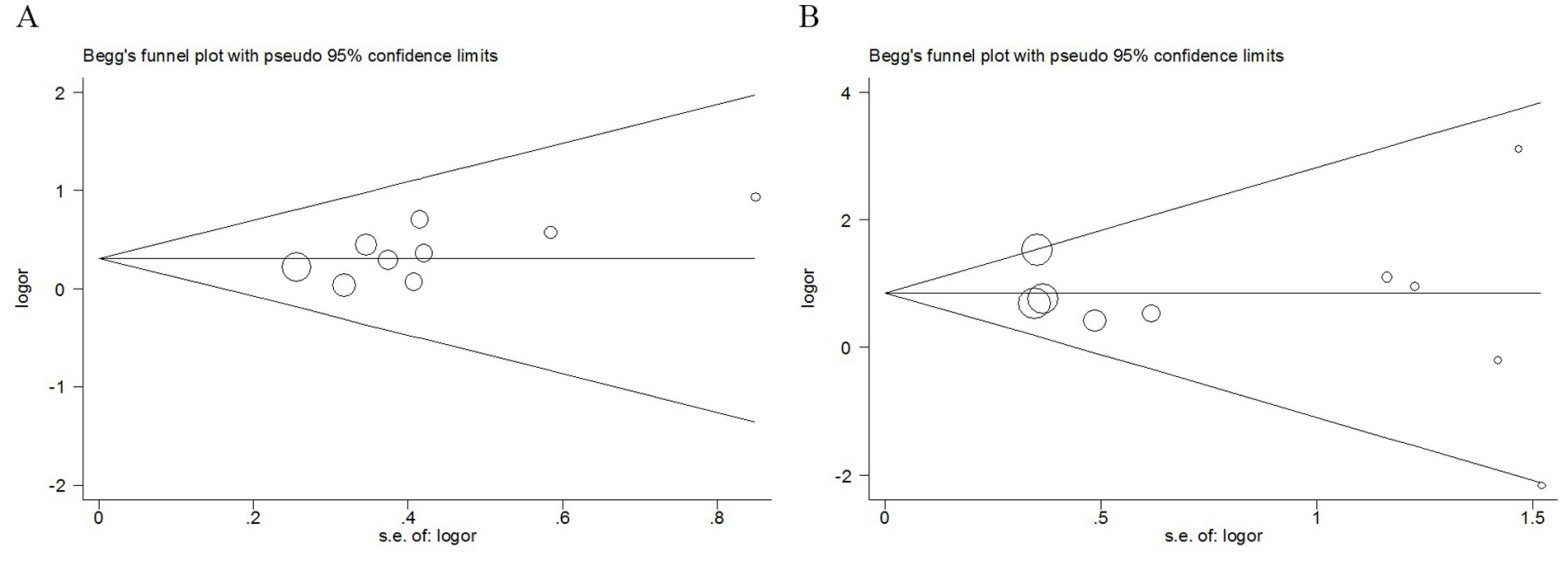
Begg’s funnel plot for publication bias test of LMP polymorphisms: rs17587 **(A)**, rs2071543 **(B)**, under homozygous model.

We also performed Begg’s and Egger’s test to assess the possible publication bias. The funnel plots of Begg’s test were symmetrical inverted (Figure 5A–5B), which suggested no significant publication bias. In addition, the results Egger’s test also showed no evidence of publication bias for LMP2/LMP7 polymorphisms. Therefore, the outcomes revealed that publication bias was not significant in this meta-analysis.

## DISCUSSION

Cancer cells can escape immune recognition via insufficient expression of peptides presented by the MHC, because HLA-mediated presentation of immunogenic cancer peptides class I is a prerequisite of a successful antitumor immune response [17]. Recent studies indicated a participation of the antigen processing machinery in carcinogenesis, such as in esophagus cancer [18], malignant melanoma [19], and hepatocellular carcinoma [20]. Two groups of proteins that participate in the antigen processing are LMPs and TAPs. Genetic polymorphisms of immunoproteasome subunits (LMP2, LMP7) and of transporter subunits (TAP1 and TAP2) are documented [21-23].

As a significant cause of population diversity, SNP is the most common type of human genetic variation correlated with cancer susceptibility [24]. Recently, much effort has been directed toward illuminating the role of LMP gene polymorphisms and their impacts on susceptibility to and progression of various diseases [7-16, 25]. Clarifying the association between LMP gene SNPs and cancer risk will help further illuminate the mechanisms underlying the carcinogenesis, which will in turn provide novel biomarkers for screening high-risk populations for cancer and improve the development of molecular-targeted therapy.

The current study is the very first meta-analysis of the association between LMP2/LMP7 polymorphisms and cancer risk. Analysis among all subjects suggested a significant increase in the risk of cancer associated with LMP2/LMP7. In our meta-analysis, the genetic heterogeneity between the selected studies was evaluated, and no significant heterogeneity was observed. All studies included were found homogeneous without any study disproportionately driving the combined estimates. In subgroup analysis of ethnicity, our results showed that both rs17587 and rs2071543 were correlated with cancer risk in Asian populations, but not in Caucasian populations. The differences between Asians and other races may be partly attribute to different genetic backgrounds, lifestyles or environments. Besides, when the stratification analyses were conducted by cancer types, we identified a significantly increased susceptibility to gynecological cancer for rs17587 polymorphism, to gastrointestinal and gynecological cancer for rs2071543.

Meta-analysis is a powerful method for analyzing cumulative data of studies where the single sample size is small and the statistical power is low [26]. However, several limitations of this meta-analysis should be acknowledged. To begin with, this analysis was based on unadjusted ORs because of a lack of information for several potential confounding variables. Secondly, due to the limited scope of databases, we cannot exclude the possibility of missing data. Additionally, more studies from all over the world should be performed to make our conclusions more persuasive.

Generally speaking, our meta-analysis manifests that LMP2/LMP7 polymorphisms (rs17587, rs2071543) are risk factors for cancer, and presence of the two polymorphisms in Asian population will increase their susceptibility to cancer. However, larger-scale multicenter studies are needed to further clarify the possible roles of the polymorphisms in the development of cancers.

## MATERIALS AND METHODS

### Primary search strategy

The PubMed, Web of Science, Google Scholar and the China National Knowledge Infrastructure (CNKI) databases were searched for relevant studies up to April 14, 2017 in both English and Chinese through with the following terms and their combinations: “LMP2”, “LMP7”, “low molecular mass protein”, “polymorphism” or “variant”, “rs17587”, “rs2071543” and “cancer” or “tumor”. The reference lists of the retrieved studies were also screened to prevent the loss of any important data. All studies involved had to satisfy the following criteria: (a) case-control design was utilized; (b) studies focused on the association of LMP2/LMP7 polymorphisms with the risk of cancer; (c) published data must have been sufficient to allow OR estimation an odds ratio (OR) with a 95% CI. (d) for publications reporting the same data or overlapping data, only the largest or latest one was selected.

### Data extraction

Initially, all the following information was extracted independently by three investigators (Y Wu, D Liu and J Zhang) and recorded in a standardized form. We extracted the following items from each article: including: first author’s name, year of publication, ethnicity of each study population, genotyping method, source of controls, cancer types, number of cancer cases and controls, allele frequencies, genotype distributions of LMP2/LMP7 polymorphisms, and Hardy-Weinberg equilibrium (HWE) results (Table 1). Disagreements were resolved through discussions involving a senior investigator (K Jiang).

### Quality assessment

The studies quality was evaluated utilizing Newcastle-Ottawa Scale (NOS) for nonrandomized studies, including case-control and cohort studies [27]. NOS awards eight points to each case-control study (four for quality of selection, one for comparability, and three for quality of exposure). A study can be awarded a maximum of one star for each point within the selection and exposure categories, and a maximum of two stars for comparability. We considered studies with scores of more than 7 as high-quality studies, and those with scores of 7 or less as low-quality studies.

### Statistical analysis

All analyses were performed using the Stata software, version 12.0 (Stata Corp., College Station, TX, USA). All P values were 2-sided and a P value <0.5 was regarded as statistically significant.

To evaluate the cancer risk associated with LMP2/LMP7 polymorphisms, the pooled odds ratios (ORs) and 95% CIs were calculated. Heterogeneity between studies was evaluated using the *I*^*2*^ test, with a higher *I*^*2*^ value indicating a higher level of heterogeneity (*I*^*2*^ = 0-25%: no heterogeneity; *I*^*2*^ = 25-50%: moderate heterogeneity; *I*^*2*^ = 50-75%: great heterogeneity; *I*^*2*^ = 75-100%: extreme heterogeneity;). Meanwhile, if heterogeneity P value was higher than 0.10, the fixed effects model (the Mantel-Haenszel method) was used [28]. Otherwise, the random-effects model (The DerSimonian-Laird method) was used [29]. Then, we performed the stratified analyses according to ethnicity, source of controls and cancer types.

A sensitivity analysis was performed to assess the stability of final results by calculating the outcomes again by omitting one single study at a time. Begg’s funnel plots and Egger’s linear regression were adopted to evaluate the publication bias [30]. HWE was checked by the goodness-of-fit chi-square test and a P < 0.05 was regarded as statistically significant [31].
